# Intrapersonal and Interpersonal Functions as Pathways to Future Self-Harm Repetition and Suicide Attempts

**DOI:** 10.3389/fpsyg.2021.688472

**Published:** 2021-07-19

**Authors:** Kathryn Jane Gardner, Elise Paul, Edward A. Selby, E. David Klonsky, Becky Mars

**Affiliations:** ^1^School of Psychology, University of Central Lancashire, Preston, United Kingdom; ^2^Research Department of Behavioural Science and Health, Institute of Epidemiology & Health, University College London, London, United Kingdom; ^3^Department of Psychology, Rutgers University, Piscataway, NJ, United States; ^4^Department of Psychology, University of British Columbia, Vancouver, BC, Canada; ^5^National Institute for Health Research (NIHR) Biomedical Research Centre, University Hospitals Bristol NHS Foundation Trust, Centre for Academic Mental Health, Population Health Sciences, University of Bristol Medical School, Bristol, United Kingdom

**Keywords:** Avon Longitudinal Study of Parents and Children, self-harm, non-suicidal self-injury, non-suicidal self-harm, suicide attempt, non-suicidal self-harm functions

## Abstract

**Background:** Research has identified functions of non-suicidal self-harm/self-injury (NSSH) but whether functions change over time, from adolescence to early adulthood, or predict the continuation of the behavior prospectively remains unclear. This study aimed to prospectively explore whether intrapersonal and interpersonal NSSH functions in adolescence predict repetition of self-harm (regardless of suicidal intent) and incident suicide attempts in early adulthood.

**Methods:** Participants were 528 individuals with NSSH at age 16 years from the Avon Longitudinal Study of Parents and Children (ALSPAC), a population-based birth cohort in the UK. Descriptive statistics were used to explore changes in functions over time from age 16 to 21, and logistic regression used to examine associations between NSSH functions and repeat self-harm and suicide attempts at age 21, 24, and 25 years.

**Findings:** The majority of 16-year-olds with NSSH endorsed intrapersonal (e.g., affect regulatory) functions only (73% at 16 years and 64% at 21 years). Just under half of adolescents (42%) and three quarters of 21 years olds reported more than one function simultaneously. A greater number of intrapersonal functions at 16 years independently predicted future repetition of self-harm at ages 21–25 years, over and above interpersonal functions (OR = 1.46, 95% CI 1.06–2.01). Interpersonal functions during adolescence did not predict repeat self-harm or suicide attempts in adulthood.

**Discussion:** Our findings suggest that intrapersonal but not interpersonal NSSH functions are a prospective risk factor for future self-harm and might also predict incident suicide attempts. The results highlight the central role of underlying affective difficulties and motivations in self-harm maintenance.

## Introduction

Self-harm encompasses both non-suicidal and suicidal behaviors and is a major risk factor for future suicide attempts (Ribeiro et al., 2016; National Confidential Inquiry into Suicide Safety in Mental Health, 2018; Mars et al., 2019) and poor mental health/well-being (Jacobson and Gould, 2007; Mars et al., 2014a). “Self-harm” is defined as any deliberate self-poisoning or self-injury to the body (e.g., cutting) irrespective of degree of suicidal intent (Hawton et al., 2003), and has a peak incidence in adolescence (Geulayov et al., 2018). This definition of self-harm does not separate suicidal from “non-suicidal self-harm” (NSSH i.e., self-harm that includes both direct self-injury and self-poisoning without suicidal intent) nor from “non-suicidal self-injury” (NSSI i.e., self-harm which excludes self-poisoning and is defined as the intentional destruction of one's own body tissue without suicidal intent and for purposes not socially sanctioned: American Psychiatric Association, 2013). In this paper, we use the broader term “NSSH” to refer to any self-harm that occurs without suicidal intent but recognize that the specific definition used may vary across studies. Notably, like self-harm, NSSH is higher in adolescence (international pooled prevalence of 17.2% compared to 13.4% for young adults and 5.5% for adults: Swannell et al., 2014), highlighting the need to identify factors that should be key targets for prevention and/or early intervention.

One factor that has received increasing attention is *why* people self-harm, that is, the functions that NSSH serves. There are many specific functions of NSSH and empirical evidence suggests that these specific functions fall broadly within two conceptually distinct categories (e.g., Klonsky et al., 2015): intrapersonal functions or reinforcement where the focus is on self (e.g., self-punishment; feeling generation/anti-dissociation; and regulating affect, the most commonly reported function; Klonsky, 2007, 2009), and interpersonal functions or reinforcement where the focus is on others (e.g., interpersonal influence; peer bonding; and seeking support/care, consistent with the “cry of pain” model; Nock, 2008). A wealth of studies over the past decade have extended our understanding of these functions (e.g., Selby et al., 2014; Taylor et al., 2018), and as a result, we now know a number of things that can inform our conceptualisations and work: (1) intrapersonal affect regulatory functions such as “releasing emotional pressures” are well-documented (Wolff et al., 2019) and tend to be the primary function of NSSH (Klonsky, 2009), which means that NSSH can be understood largely from the perspective of emotion regulation/dysregulation (Chapman et al., 2006; Andover and Morris, 2014); (2) intrapersonal and interpersonal functions can be positioned within broader theoretical models of NSSH as two maintaining and reinforcing routes to NSSH (Nock, 2009, 2010), but are not mutually exclusive. Indeed, studies have shown that most people simultaneously endorse multiple functions of NSSH within both domains (e.g., Klonsky and Glenn, 2009; Klonsky, 2011). Whilst however, these functions are “non-suicidal,” they also predict suicidal outcomes such as suicide attempts (e.g., Roley-Roberts et al., 2017). This association between NSSH functions and suicide attempts can be understood in terms of common mechanisms/risk factors (e.g., emotion distress/dysregulation and affective disorders: Hamza et al., 2012; Mars et al., 2014b; Victor and Klonsky, 2014; Law et al., 2015; Grandclerc et al., 2016). Alternatively, individuals who engage in NSSH develop capability for suicide through habituation to pain and fear (Joiner et al., 2012; Klonsky et al., 2013). As we describe below, there are a large number of cross-sectional studies of functions in relation to both specific aspects of NSSH behavior, and suicidality. Yet, there are gaps with only a handful of studies *prospectively* examining the extent to which functions predict future NSSH repetition over time, and there are to the best of our knowledge no prospective studies that have examined how NSSH functions predict incident suicide attempts.

Cross-sectional studies of associations between NSSH functions and NSSH behavior have examined characteristics such as method, frequency and severity of NSSH. Studies have found that intrapersonal relative to interpersonal functions better predict life-time frequency of NSSH (e.g., Saraff et al., 2015), more clinically severe NSSH (greater current frequency of NSSH and urges; Klonsky et al., 2015), and retrospective reports of continued engagement in NSSH from adolescence to adulthood (Halpin and Duffy, 2020). Associations for interpersonal functions are typically, though not always, smaller, and there is evidence also that the need to self-harm for interpersonal reasons might be time-limited and restricted since these functions increase the likelihood of NSSH cessation from adolescence to adulthood (Halpin and Duffy, 2020). It seems therefore, that when NSSH does operate as an interpersonal behavioral coping strategy that this is usually during adolescence, perhaps in response to the complex social and relational challenges faced by adolescents during this period of development. Consistent with this, Muehlenkamp et al. (2013) found that interpersonal functions are more commonly endorsed for *initiating* NSSH (which typically happens during adolescence), whilst intrapersonal functions are more likely to underpin self-reported *repeated* NSSH. In comparison to interpersonal functions therefore, intrapersonal functions might better maintain NSSH over time. Further support for the reinforcing/maintaining effects of intrapersonal functions comes from studies showing that individuals who more frequently self-injure experience the most benefits in terms of reduced negative affect (e.g., Klonsky, 2009), and perceive NSSH as being effective in meeting their intrapersonal needs (Brausch and Muehlenkamp, 2018). Taken together, the evidence from cross-sectional studies of NSSH functions and behavior supports an affect regulation perspective (Chapman et al., 2006; Andover and Morris, 2014) rather than social signaling hypothesis (Nock, 2008) of NSSH maintenance/repetition, and highlights potential changes in the reasons why people engage in NSSH over time i.e., interpersonal functions are typically most prominent during adolescence whilst intrapersonal persist across adolescence and adulthood.

The empirical association between NSSH functions and suicidality (ideation and past attempts) has also been explored throughout many cross-sectional studies, typically of University/College students. These studies also highlight the relative importance of intrapersonal functions for aspects of suicidality (Klonsky and Olino, 2008; Paul et al., 2015; Roley-Roberts et al., 2017; Brausch and Muehlenkamp, 2018; O'Loughlin et al., 2020), though there is variation in effect sizes. For example, Klonsky and Glenn (2009) found small associations between suicide attempts and both intrapersonal and interpersonal function domains, but suicidal ideation was more strongly associated with intrapersonal than interpersonal functions. Ultimately, the patterns across most studies in nonclinical adults suggests that intrapersonal functions may heighten the risk for a more imminent engagement in suicide attempts (e.g., O'Loughlin et al., 2020) but that interpersonal functions could also be important. There are fewer studies of functions in adolescents [see Taylor et al. (2018), for review], and of the studies that have explored functions in relation to suicide, the findings are also mixed (e.g., Nock and Prinstein, 2005; Lloyd-Richardson et al., 2007).

Unfortunately, a key limitation of cross-sectional studies is the reliance on retrospective reports of NSSH behavior and characteristics/functions and suicide-related outcomes. Only a small handful of longitudinal studies have examined whether NSSH functions predict future NSSH repetition, finding also that intrapersonal functions are key to repetition. Yet, these studies use relatively short-time periods and/or small samples. Glenn and Klonsky (2011a) found that neither intrapersonal nor interpersonal functions prospectively predicted the frequency (repetition) of NSSH at 12 months in a sample of 51 students, though the small sample renders conclusions tentative. In a high risk inpatient sample of 40 adolescents, intra- but not interpersonal functions are associated with NSSH maintenance over 6 months (Yen et al., 2016), an effect that has been replicated in a 3-year longitudinal study of 51 students from late adolescence to early adulthood (Kiekens et al., 2017). Finally, in a clinical sample of 262 adults with Borderline Personality Disorder followed up every 2 years over a 16-year period, intra- but not interpersonal reasons were significantly more likely to be reported by those with more extensive self-harm (Zanarini et al., 2013). To the best of our knowledge therefore, only one non-clinical study in this area (Kiekens et al., 2017) has examined how functions predict self-harm outcomes over at least several years and during the period of adolescence to adulthood, yet the sample size was small. Moreover, with regards to NSSH functions and suicidal behavior, to the best of our knowledge there are no prospective studies examining whether NSSH intra- and interpersonal functions predict first-time suicide attempts among those with NSSH. Such studies can help us understand who, from those who engage in NSSH, are more at risk of making subsequent suicide attempts. In sum, longitudinal work to date suggests that intrapersonal NSSH functions might better maintain NSSH. Yet, long-time prospective studies (i.e., >3 years) of NSSH functions and self-harm/suicidal outcomes that use large samples are needed to clarify the nature of these associations over time, especially from adolescence—when NSSH is more likely to be initiated—through to adulthood.

Another gap in the literature relates to longitudinal studies of stability or changes in functions over long time periods, from adolescence to adulthood. Understanding stability in functions (or lack thereof) is important for continued refinement of theoretical models (Nock, 2009, 2010) which currently do not delineate changes in the reinforcing properties of functions over time; and second, for contextualizing prospective associations between NSSH functions and NSSH behavior/suicide attempts. For example, if intrapersonal functions maintain self-harm, then we'd simultaneously expect some degree of stability in functions over time. There is some albeit limited longitudinal work here, with studies of University students (Glenn and Klonsky, 2011b) and clinical samples (Victor et al., 2016; Daukantaite et al., 2020; Pérez et al., 2020) finding moderate to large stability coefficients over short time periods (<12 months) when assessing functions *via* the Inventory of Statements about Self-Injury (ISAS; Klonsky and Glenn, 2009). Whilst the size of these coefficients varies across the studies, intrapersonal functions are typically more highly endorsed at multiple time points than interpersonal ones, and might therefore better reinforce self-harm over time. ISAS (Pérez et al., 2020). Taken together, the findings from these studies suggest some degree of stability but also change in both intrapersonal and interpersonal functions over relatively short time frames. We are not aware of any long-term prospective studies examining patterns in functions over time in lower risk non-clinical samples. Such studies are an important endeavor since they can elucidate whether functions change when NSSH is potentially becoming entrenched during periods of developmental transition to adulthood, and whether they are subsequently likely to predict other outcomes over time.

In sum, whilst there exist some longitudinal studies of NSSH functions and self-harm outcomes these mostly use small samples and span short-time frames of <12-months. This study therefore extends previous research by using a large community-based cohort sample to examine the contribution of intrapersonal and interpersonal functions to self-harm outcomes during developmental transition from adolescence into early adulthood, and whether functions change over time. This contribution is important to establish on theoretical and clinical grounds, and specifically in relation to continued engagement in (i.e., repetition of) self-harm and incident suicide attempts. This study fills this gap *via* three specific objectives:

- describe the intrapersonal and interpersonal functions of self-harm at age 16 and 21 and examine how they change over these two time points.
- explore whether the number of NSSH intrapersonal and interpersonal functions at age 16 years predicts continued engagement in/repetition of self-harm in young adulthood.
- explore whether the total number of NSSH intrapersonal and interpersonal functions at age 16 years predicts future incident suicide attempts (from age 16 to age 25 years).

## Methods

### Sample

The Avon Longitudinal Study of Parents and Children (ALSPAC) is an ongoing population-based birth cohort study examining influences on health and development across the life-course. The ALSPAC core enrolled sample consists of 14,541 pregnant women residing in the former county of Avon in South West England (UK), with expected delivery dates between 1st April 1991 and 31st December 1992 (Boyd et al., 2013; Fraser et al., 2013; Northstone et al., 2019). Of the 14,062 live births in the core sample, 13,798 were singletons/first-born of twins and were alive at 1 year of age. Participants have been followed-up regularly since recruitment through questionnaires and research clinics. The study website contains details of all the data that is available through a fully searchable data dictionary http://www.bris.ac.uk/alspac/researchers/our-data. Ethical approval for the study was obtained from the ALSPAC Ethics and Law Committee and the Local Research Ethics Committees. Study data were collected and managed using REDCap electronic data capture tools hosted at University of Bristol (Harris et al., 2009, 2019).

The present investigation is based on the subsample of participants who completed a detailed self-report questionnaire on self-harm at age 16 years (*N* = 4,806), and who were then followed over three additional waves of data collection at ages 21, 24, and 25 years. Self-harm was assessed with the question: “Have you ever hurt yourself on purpose in any way (e.g., by taking an overdose of pills, or by cutting yourself)?” which was endorsed by 905 (18.8%) participants. As our interest was in functions for NSSH, those who reported they had ever attempted suicide at age 16 years were excluded from the analysis (*n* = 325). This also enabled us to investigate the relationship between NSSH functions at baseline and first-time suicide attempts at follow-up. The number of participants with NSSH at age 16 years (who had never made a suicide attempt) and who had data on self-harm functions was 528, after excluding 41 participants with missing data on self-harm functions and 11 with missing data on suicidal intent.

### Measures

#### Predictor: Self-Harm Functions

At ages 16- and 21-years, young people who said they had self-harmed were asked to select the reason(s) for their most recent self-harm episode from a pre-defined list of six options. Response options included “to show how desperate I was feeling;” “I wanted to die;” “to punish myself;” “to frighten someone;” “to gain relief from a terrible state of mind;” and “other reason.” Those who selected “other reason” were asked to specify their motivation(s) using a free text response. These free-text responses were then categorized into themes by BM. There were 18 additional response categories identified at age 16 years and 16 additional categories at age 21 years. Participants were able to select more than one response option. Each function was coded as present or absent and summed to give (a) the total number of functions, (b) the total number of intrapersonal functions, and (c) the total number of interpersonal functions. See Supplementary Table 1 for a full list of functions. At each time point, participants who did not select a reason for their self-harm, provided the response “I don't know,” or selected a reason endorsed by fewer than five participants (out of the 905 who had self-harmed) were coded as missing (*n* = 41 at 16 years and *n* = 2 at 21 years). This step was necessary to comply with ALSPAC confidentiality rules. In addition, as our analysis focused on NSSH functions at age 16 years, participants who selected “I wanted to die” as a reason for their most recent self-harm episode at 16 years were excluded from the analysis. Data on self-harm functions was not recorded at age 24 or 25 years.

#### Outcome Measures: Past Year Self-Harm and New Onset Suicidal Self-Harm

Self-harm was assessed *via* self-report at ages 21, 24, and 25 years. Participants were sent an online/postal questionnaire at ages 21 and 25 years and were invited to attend a research clinic at 24 years. The questions were based on those used in the Child and Adolescent Self-harm in Europe (CASE) study (Madge et al., 2008). Each time, participants were asked an initial screen question “Have you ever hurt yourself on purpose in any way (e.g., by taking an overdose of pills, or by cutting yourself)?” Response options were Yes or No. Those who responded positively were then asked a series of follow-up questions to assess past year self-harm frequency and presence of suicidal intent. Past year frequency was recoded into a binary presence/absence variable (0 = no past year self-harm; 1 = past year self-harm) and incudes those who have self-harmed with or without suicidal intent.

Participants were classified as having ever attempted suicide if they: (a) selected “I wanted to die” as a reason for self-harm (asked at ages 16, 21, and 25); or (b) answered “yes” to: “On any of the occasions when you have hurt yourself on purpose, have you ever seriously wanted to kill yourself?” (asked at all time points). Suicide attempts were assessed in the same way at age 16 years. As those who had self-harmed with suicidal intent at age 16 years were excluded from the analysis, the lifetime suicidal self-harm measure at follow-up refers to incident suicide attempts occurring after the age of 16 years.

Response options from the three follow-up periods were then combined to generate two outcome variables: (1) any repeat self-harm during follow up (past year self-harm reported at any time point at age 21, 24, or 25 years), and (2) incident suicide attempt during follow up (lifetime suicide attempt since age 16 reported at age 21, 24, or 25 years).

#### Covariates

Covariates were child sex and two measures of socioeconomic position- maternal education level shortly after birth (O levels or lower versus A levels or higher) and income quintiles. Income was assessed *via* maternal questionnaire and included average weekly household disposable income recorded at age 3 and 4 years, divided into quintiles and rescaled to account for family size, composition, and estimated housing benefits (Gregg et al., 2008).

### Statistical Analysis Plan

We first report descriptive data on changes in NSSH functions over time using complete case data. All main (outcome) analysis was imputed and used logistic regression to examine associations between NSSH functions at age 16 years (total number of functions, number of intrapersonal functions, and number of interpersonal functions) and the two self-harm outcomes: repeat self-harm and suicide attempts reported at age 21–25 years using imputed data (see below for details). Analysis models of interpersonal/intrapersonal functions were mutually adjusted for each other (Model 1). Analyses were also adjusted for relevant confounders (Model 2). Unadjusted results are provided for comparison.

#### Missing Data

The main analyses looking at self-harm outcomes were conducted on an imputed dataset based on those who had data on self-harm functions at 16 years (*N* = 528). The number with complete data (combined self-harm outcome data and information on all confounders) was 198 for repeat self-harm and 192 for suicide attempts. The proportion with missing outcome data for past year self-harm at each time point was 33.5% at age 21, 39.0% at 24 and 36.6% at 25 years. The proportion with missing outcome data for lifetime suicide attempts at each time point was 33.0% at age 21, 39.0% at 24 and 39.2% at 25 years. Missing outcome and confounder data were imputed using Multiple Imputation by Chained Equations (MICE; Royston and White, 2011). One hundred imputed datasets were generated. The imputation model incorporated all variables used in the analyses as well as relevant auxiliary variables (e.g., socioeconomic status, mental health outcomes, substance use, and earlier or later recordings of variables of interest). This method assumes that data are missing at random (MAR), whereby any systematic differences between the missing and the observed values can be explained by differences in observed data. All analyses were conducted using Stata version 15. Outcome data were imputed for each point separately and then combined in each dataset as detailed previously. The OR estimates were broadly consistent across the compete case and imputed datasets, however the complete case data are less precise due to the smaller sample size (Supplementary Table 2).

## Results

Table 1 shows the self-harm functions endorsed by participants at ages 16 and 21 years in the complete case sample (descriptive statistics for sample demographics use imputed data and therefore appear in **Table 3** with the main analyses). Of the 528 participants who had engaged in NSSH at 16 years, 488 (92.4%) reported at least one intrapersonal function and 143 (27.1%) reported at least one interpersonal function. Only 7.6% of the sample reported interpersonal functions only, with most participants reporting either intrapersonal functions only (72.9%) or both types (19.5%). Thus, 92% reported some form of intrapersonal function. At age 16 years, 58% endorsed only one function and the remaining 42% endorsed two, three or in some cases more NSSH functions simultaneously.

**Table 1:** Comparison of self-harm functions at 16 and 21 years: complete case data.

	**Self-harm functions at age 16 years *N* = 528**	**Self-harm functions at age 21 years *N* = 59**
**All functions**		
*Total number, median (IQR)*	1 (1, 2)	2 (1, 3)
*One function*	306 (58.0%)	15 (25.4%)
*Two functions*	155 (29.4%)	19 (32.2%)
*Three or more functions*	53 (10.0%)	15 (25.4%)
*Four or more functions*	14 (2.7%)	10 (17.0%)
**Intrapersonal functions**		
*Total number, median (IQR)*	1 (1, 2)	2 (1, 3)
*Zero functions*	40 (7.6%)	N/A
*One function*	328 (62.1%)	21 (35.6%)
*Two functions*	137 (26.0%)	23 (39.0%)
*Three or more functions*	23 (4.3%)	15 (25.4%)
**Interpersonal functions**		
*Total number, median (IQR)*	0 (0, 1)	0 (0, 1)
*Zero functions*	385 (72.9%)	38 (64.4%)
*One function*	127 (24.1%)	16 (27.1%)
*Two or more functions*	16 (3.0%)	5 (8.5%)
Intrapersonal functions only	385 (72.9%)	38 (64.4%)
Interpersonal functions only	40 (7.6%)	N/A
Both intra- and interpersonal functions	103 (19.5%)	21 (35.6%)

*Age 21 includes the self-harm function “I wanted to die”. Participants who endorsed this function at age 16 years were excluded from the analysis*.

Data on self-harm at age 21 years was available for 351 out of the 528 who reported self-harm at age 16 years (66.5%). Of these, 61 reported past year self-harm at 21, and information on functions was available for 59 individuals. All 59 reported at least one intrapersonal function and 21 (35.6%) reported at least one interpersonal function. Most participants reported intrapersonal functions only (64.4%) with the remainder reporting both types (35.6%). Thus, 100% reported some form of intrapersonal function. At age 21 years, 25.4% endorsed only one function and the remaining 74.6% multiple self-harm functions.

### Changes in NSSH Functions Over Time

Table 2 shows changes in self-harm functions between 16 and 21 years for the 59 participants who self-harmed in the past year at age 21 and had data on self-harm functions (referring to the most recent episode). Of those who reported only intrapersonal functions at age 16, the majority (68.9%) still reported intrapersonal functions only at age 21. Thirty-one percent reported either interpersonal functions only, or both types at 21 years (n.b. these categories were combined due to low cell counts). Of those who reported either interpersonal only or both types at 16 years, half switched to intrapersonal only at age 21.

**Table 2:** Proportions of Intrapersonal and Interpersonal NSSH functions at age 16 and 21.

**Age 16 functions**	**Age 21 functions**	
	**Intrapersonal only**	**Interpersonal only/both**
Intrapersonal only (*n* = 45)	31 (68.9%)	14 (31.1%)
Interpersonal only/both (*n* = 14)	7 (50%)	7 (50%)
	**At least one intrapersonal**	**At least one interpersonal**
At least one intrapersonal (*n* = 57)	57 (100%)	20 (35.1%)
At least one interpersonal (*n* = 14)	14 (100%)	7 (50%)

*Chi-square could not be computed since these categories are not mutually exclusive/from a single cross-tab i.e., individuals who reported at least one intrapersonal function can also report interpersonal, and vice versa*.

All participants reported at least one intrapersonal function at age 21 years. Participants were more likely to endorse an interpersonal function at 21 years if they had reported at least one interpersonal function at baseline (50% compared to 35.1% among those who reported at least one intrapersonal function at 16).

### Association Between Number of NSSH Functions and Future Repeated Self-Harm: Imputed Data (*N* = 528)

The proportion of the sample who reported repeat self-harm (past year self-harm at 21, 24, or 25 years) was 33.5% (95% CI 28.3–38.6%). At follow-up, nearly one-third (29.2%; 95% CI 23.8–34.5%) reported having attempted suicide for the first time since age 16 years. Table 3 shows the sociodemographic and NSSH function characteristics at baseline for different outcome variables. Table 4 shows the results of the logistic regression analysis between NSSH functions at 16 and future self-harm and suicide attempts.

**Table 3:** Sociodemographic and NSSH function characteristics at baseline according to self-harm outcome: imputed data.

	**Participants with NSSH at 16 years *N* = 528**	**Participants with repeat self-harm at follow-up (past year)**	**Participants with new onset suicide attempt at follow-up**
	**M (SE) or %**	**M (SE) or %**	**M (SE) or %**
Female sex	79.9%	79.9%	76.3%
**Maternal education (missing data)**			
*O-Levels or lower*	52.0%	46.0%	54.1%
*A-Levels or higher*	48.0%	54.0%	45.9%
**Family income quintiles**			
*1st*	14.1%	16.9%	9.6%
*2nd*	17.7%	19.8%	24.4%
*3rd*	21.5%	18.1%	19.5%
*4th*	22.8%	18.5%	20.7%
*5th*	23.9%	26.7%	25.8%
**NSSH functions at age 16 years**			
All functions			
*Total number, median (IQR)*	1 (1, 2)	1 (1, 2)	1 (1, 2)
*One function*	58.0%	53.1%	51.6%
*Two functions*	29.4%	30.0%	28.1%
*Three or more functions*	12.7%	16.9%	20.4%
Intrapersonal functions			
*Total number, median (IQR)*	1 (1, 2)	1 (1, 2)	1 (1, 2)
*Zero functions*	7.6%	4.4%	3.9%
*One function*	62.1%	60.4%	59.5%
*Two functions*	26.0%	28.2%	29.3%
*Three or more functions*	4.4%	6.9%	7.31%
Interpersonal functions			
*Total number, median (IQR)*	0 (0, 1)	0 (0, 1)	0 (0, 1)
*Zero functions*	72.9%	73.9%	71.8%
*One or more functions*	27.1%	26.1%	28.2%
Intrapersonal functions only	72.9%	73.9%	71.8%
Interpersonal functions only	7.6%	4.4%	3.9%
Both intra- and interpersonal functions	19.5%	21.7%	24.3%

*Responses reflect self-harm functions for last time young person self-harmed*.

*Non-suicidal self-harm functions were measured at age 16*.

*Sample sizes at follow-up are not available as data are imputed*.

**Table 4:** Functions of NSSH as predictors of future self-harm and suicide attempts: imputed data.

	**Unadjusted**		**Model 1**		**Model 2**	
	**OR (95%CI)**	***P*-value**	**OR (95%CI)**	***P*-value**	**OR (95%CI)**	***P*-value**
**Repeat self-harm**						
Total functions	1.39 (1.07, 1.81)	0.014	-	-	1.40 (1.07, 1.84)	0.015
Intrapersonal functions	1.43 (1.04, 1.96)	0.028	1.46 (1.07, 2.00)	0.018	1.46 (1.06, 2.01)	0.021
Interpersonal functions	1.19 (0.78, 1.81)	0.419	1.28 (0.83, 1.96)	0.265	1.30 (0.85, 2.01)	0.230
**New onset suicidal self-harm**						
Total functions	1.24 (0.92, 1.67)	0.152	-	-	1.28 (0.95, 1.74)	0.108
Intrapersonal functions	1.32 (0.92, 1.88)	0.130	1.33 (0.93, 1.90)	0.119	1.36 (0.94, 1.97)	0.101
Interpersonal functions	1.04 (0.67, 1.62)	0.863	1.09 (0.70, 1.72)	0.699	1.15 (0.73, 1.82)	0.548

*Model 1: Included both intrapersonal and interpersonal functions. Model 2: Adjusted for sex, maternal education, and household income*.

#### Repeat Self-Harm

In fully adjusted models, there was strong evidence for an association between total number of NSSH functions at 16 years and future repetition of self-harm at ages 21–25 years (adjusted OR = 1.40, 95% CI 1.07, 1.84). The odds of repetition were higher among those participants who endorsed a greater number of intrapersonal functions at 16 years (adjusted OR = 1.46, 95% CI 1.06, 2.01), but we did not find an association with interpersonal functions (adjusted OR = 1.30, 95% CI 0.85, 2.01).

#### Future Suicide Attempts

In fully adjusted models, there was weak evidence for an association between the total number of NSSH functions (adjusted OR = 1.28, 95% CI 0.95, 1.74), and the total number of intrapersonal functions (adjusted OR = 1.36, 95% CI 0.94, 1.97) reported at age 16 years and future suicide attempts (findings do not reach conventional levels of significance). We did not find evidence for an association with interpersonal functions (adjusted OR = 1.15, 95% CI 0.73, 1.82) with suicide attempt.

## Discussion

Whilst many studies have empirically examined associations between intra- and interpersonal self-harm functions and how they relate to self-harm and suicidal outcomes, few have done this longitudinally nor during periods of developmental transition. This study elucidates whether NSSH functions change over time within individuals and clarifies the nature of the association between NSSH functions in adolescence and future self-harm and suicide attempts in early adulthood using a prospective cohort study.

Regarding the endorsement of *any* specific intra- and/or interpersonal self-harm function at age 16 and 21, we found that 42% simultaneously endorsed multiple (usually two or three) specific functions during adolescence and this increased to 74.6% during adulthood. This pattern is consistent with studies of adults and adolescents that have used broader validated measures of NSSH functions such as the ISAS (Klonsky and Glenn, 2009) or FASM (Functional Assessment of Self-Mutilation; Lloyd-Richardson et al., 2007) where the number of functions seems to be higher in adulthood [e.g., Nock and Prinstein, 2005; Lloyd-Richardson et al., 2007; Klonsky, 2011; see also the meta-analysis by Taylor et al. (2018)]. The pattern suggests that individuals might discover more specific functions for NSSH over time, though our discrepancy in function endorsement during adolescence and adulthood may be due in part to sample characteristics. In adulthood, our focus was on a smaller number who reported repeat self-harm in the previous year at age 21 years. We also included one additional “suicidal function” item (“I wanted to die”) at 21 years which was reported by 14 (23.7%) participants. Those who endorsed this function at age 16 were excluded to ensure our study sample only contained those who had harmed without suicidal intent at baseline, but excluding these individuals could have more generally reduced the number of functions at baseline (cross-sectional work suggests that the number of functions correlates positively with past suicide attempts i.e., there are on average more functions present in those who have attempted suicide; e.g., Klonsky and Glenn, 2009). We are likely therefore to be capturing adults with more chronic and entrenched self-harm.

When comparing the patterns of intrapersonal and interpersonal functions, we found that 92% of adolescents and 100% of adults endorsed at least one specific intrapersonal function (alone or alongside interpersonal functions). Similarly high percentages have been reported in some previous non-clinical samples (e.g., Saraff and Pepper, 2014), though the pooled prevalence of intrapersonal functions across a range of sample types is slightly lower at 66–81% (interpersonal functions is lower still at 33–56%: Taylor et al., 2018). More frequent endorsement of intrapersonal functions at both time points is also consistent with previous studies of stability over 12 months (Glenn and Klonsky, 2011b; Daukantaite et al., 2020). This pattern is important to understand because more frequently endorsed stable functions might better reinforce self-harm over time.

We also found that endorsing both types of function was more common during adulthood than at 16 years, and that no adults endorsed interpersonal functions *only* compared with 7.6% during adolescence. Rather, when interpersonal functions were present in adulthood they were always accompanied by intrapersonal functions; this pattern suggests that interpersonal functions may trigger self-harm initiation during adolescence but only serve to maintain self-harm over time in the presence of intrapersonal reasons. This conclusion fits with previous work highlighting the importance of interpersonal functions for self-harm initiation, but not maintenance (Muehlenkamp et al., 2013; Tatnell et al., 2014). Moreover, our data suggests that whilst the majority (68.9%) endorse intrapersonal only during both adolescence and adulthood, for others there is a switch to fewer general types of functions (i.e., from endorsing *both* interpersonal and intrapersonal, to intrapersonal only) or an accumulation of the types of reasons as they move into adulthood (i.e., a change to endorsing intra- as well as interpersonal functions).

Through assessing the functions of NSSH during adolescence we were able to examine whether these maintain future self-harm behavior. Greater endorsement of intrapersonal NSSH functions at 16 years independently predicted future repetition of self-harm at ages 21–25 years, over and above interpersonal functions. Since intrapersonal functions are also associated with greater NSSH frequency (e.g., Saraff et al., 2015) and self-harm cessation is driven by improvements in affect regulation (Whitlock et al., 2015), it is perhaps not surprising that intrapersonal functions (which capture emotion dysregulation) predict continued engagement in self-harm. Like other cross-sectional studies (e.g., Muehlenkamp et al., 2013) and in line with our conclusions based on patterns/changes in functions over time, these results support both an emotion dysregulation perspective of self-harm maintenance (Chapman et al., 2006; Andover and Morris, 2014; Wolff et al., 2019) and Nock's (2009, 2010) theoretical model which proposes that intrapersonal functions reinforce and maintain self-harm (e.g., Nock, 2009). The notion that self-harm is maintained into adulthood because it is effective in regulating affect is supported by Brausch and Muehlenkamp's (2018) cross-sectional exploration of the relative greater perceived effectiveness of NSSH for intrapersonal functions. As Brausch and Muehlenkamp (2018) cogently explain, if NSSH is effective in meeting the desired function this can lead to increased NSSH severity (e.g., lifetime frequency) because the self-harm needs have been met and continue to be reinforced over time. We apply the same logic here: if self-harm is effective and meets intrapersonal needs (e.g., it works to regulate emotion/affect), then the behavior is repeated.

We did not find an association between the number of interpersonal functions and future repetition of self-harm. This finding is also consistent with past work that has demonstrated the centrality of interpersonal functions for self-harm initiation, but not maintenance (Muehlenkamp et al., 2013; Tatnell et al., 2014). One explanation for the lack of association over time is to do with the effectiveness of self-harm for interpersonal reasons, as discussed by Brausch and Muehlenkamp (2018). If interpersonally driven self-harm is generally wholly ineffective in achieving the intended outcome such as to “show how I am feeling,” it is therefore not reinforced. Alternatively, if it is effective, it may lead to receiving support/care, which could reduce future risk of repetition. Our conclusions are tentative here since we did not measure the effectiveness of functions. These alternative plausible explanations need empirically investigating. Our findings, taken together with Brausch and Muehlenkamp, highlight the need to reconsider the reinforcing properties of interpersonal functions outlined by Nock's (2009; 2010) model of NSSH.

Regarding incident suicide attempts, we found weak evidence for an association with intrapersonal functions (Sterne and Smith, 2001; Amrhein et al., 2019; findings did not reach conventional levels of statistical significance but could be clinically important). The relationship between intrapersonal functions and suicide attempts has been documented in a number of cross-sectional studies (e.g., Klonsky and Olino, 2008) and might be explained by common mechanisms such as emotion dysregulation, that is, intrapersonal functions are an indicator of emotion distress which increases suicide desire/ideation and attempts. Alternatively, we suggest that one way in which intrapersonal functions could be associated with suicide attempts is *via* repeat self-harm. Psychological models emphasize the importance of capability for suicide (Joiner et al., 2012) and there is evidence that repeat rather than single episode self-harm elevates risk of suicide (Zahl and Hawton, 2004; Haw et al., 2007).

Interpersonal functions during adolescence were not associated with incident suicide attempts. This is perhaps not surprising if we assume that functions do predict suicide attempts *via* repeat self-harm (the latter of which was also not associated with interpersonal functions). These results suggest instead that interpersonal functions might have limited relevance over the long-term for self-harm maintenance or clinical severity in general, including suicide risk. The notion that interpersonal functions (alone) are generally less clinically significant is supported by previous studies (e.g., Klonsky and Glenn, 2009; Klonsky et al., 2015). Yet, it is important to also determine whether the *ineffectiveness* of interpersonal functions can account for the lack of association with suicide attempts and repeat self-harm, that is, whether the self-harm is ineffective in meeting interpersonal needs and as a result the behavior is not maintained, nor risk of suicide increased. There is some suggestion from Brausch and Muehlenkamp's (2018) findings that interpersonal functions are not perceived to be immediately effective in achieving desired NSSH outcomes. More recently, Snir et al.'s (2018) analysis suggests a more complex pattern of intra- and interpersonal consequences of self-harm in adolescents: self-harm measured at 3-months predicted decreases in negative affect (intrapersonal) at 12-months for adolescents high in peer support (interpersonal), and increases in negative affect for those low in peer support. Further long-term prospective studies are needed to examine functional consequences of self-harm to elucidate whether the events and experiences that occur immediately after the injury and in the future (e.g., reduced negative affect, support from family/friends) are reinforcing. Interestingly, our patterns in functions over time also highlights the fact that interpersonal functions might only exert an influence in adulthood when accompanied by intrapersonal functions.

### Future Directions and Limitations

This is a novel study and strengths include the prospective design over a long time-period, permitting exploration of prospective associations from adolescence to adulthood, and the large population-based sample. Yet, there are some limitations. First, our data only permitted exploration of associations over time between NSSH functions at age 16 and repetition of *any self-harm* at age 21 and 25 years (i.e., both suicidal and non-suicidal combined); future research must separate these out to identify whether NSSH during adolescence predicts NSSH in adulthood. Studies here should endeavor also to extend the time period, beginning in early adolescence (age 12–14) to capture early onset self-harm since incidence in the community is also high in younger adolescents (Geulayov et al., 2018).

Second, participants were asked about their motivations related to the last time they self-harmed (which we then categorized into the two broad intrapersonal or interpersonal domains), and this may not necessarily be representative of all specific functions that are driving the self-harm for that individual. We did not explore patterns in specific functions due to small sample sizes for some functions, and it is also worth noting that there is overwhelming empirical support for the two distinct but related function domains (e.g., Klonsky et al., 2015).

Third, we excluded adolescents who had attempted suicide at age 16. This was necessary to ensure we were able to focus on functions of NSSH only, however we recognize that we will have excluded some adolescents who have engaged in both behaviors. Our findings are therefore only generalisable to those who have never attempted suicide by age 16. Findings may also not generalize to other ethnic groups, as 97% of the sample were white.

Fourth, determination of suicidal intent was based on self-report and may include bias; for example, adolescents may be ambivalent or fluctuate in their intent to die and reports may be influenced by current mood state or change over time. We found that some young people reported wanting to die on the most recent occasion but then responded negatively to the later question “have you ever seriously wanted to kill yourself” (23% at age 21 years and 16% at age 25 years). For this group, self-harm may have been an expression of distress, rather than a reflection of suicidal intention. Previous work with this cohort has found that participants who have self-harmed with suicidal intent were more likely than those with non-suicidal self-harm to use overdose as a method and to have sought help, providing some support for the distinction between the groups.

Fifth, the amalgamation of data across data collection waves means that we were not able to examine self-harm frequency, yet, studies have shown important associations between functions and frequency (e.g., Saraff et al., 2015) and therefore the potential for functions to predict progression to more frequent self-harm. Other work has shown that more NSSH functions is associated with higher NSSH frequency; thus, our finding that more NSSH functions predicts repeated NSSH might be because more NSSH functions is a proxy for higher NSSH frequency. Future longitudinal work should measure both NSSH functions and frequency to determine if each provides unique information about future NSSH.

Sixth, as with all cohort studies, there was some loss to follow-up, and it is possible that non-random response may have biased our complete case analyses. Data from simulation studies suggest that unbiased results can be obtained using multiple imputation even with large proportions of missing data (up to 90%), provided data are missing at random and the imputation model is properly specified (Madley-Dowd et al., 2019). Although we cannot say with absolute certainty that the data were missing at random, our imputation models included a wealth of auxiliary information, which increases the plausibility of the missing at random assumption.

Finally, we did not examine the potential interaction between intrapersonal and interpersonal functions. Nor did we examine other potential affective/interpersonal covariates, moderators or mediators [see Abdelraheem et al. (2019), for review] such as depression which could help to explain associations between functions over time, and/or the associations between functions and future self-harm/suicidal behavior. For example, one possibility is that repeat self-harm mediates the relationship between intrapersonal functions and suicide attempts. We did not examine this possibility in this study as data were combined across time points and a clear temporal relationship which is necessary for mediation, could not be established (repeat self-harm and suicide attempts were assessed over the same time period). Future work should also examine how NSSH functions relate to a range of distal and proximal vulnerability factors that might maintain and predict NSSH over time, providing a more comprehensive test of Nock's (2009; 2010) etiological model of NSSH. Such an endeavor is important for continued refinement of evidence-based theories that explain why people engage in and repeat self-harm.

### Theoretical and Clinical Implications

Ultimately, our findings suggest that intrapersonal functions maintain self-harm and might also elevate risk of suicide attempts, whilst interpersonal functions do not. That is, intrapersonal functions play a crucial role as self-harm is potentially becoming entrenched over time throughout adolescence to early adulthood, coinciding with a period of significant adjustment where normative development involves the learning of adaptive emotion regulation skills (Gullone et al., 2010). These findings extend previous cross-sectional and prospective work regarding the reinforcing mechanisms of self-harm and with replication would suggest the need to refine existing models of NSSH (i.e., Nock, 2009, 2010) to capture changes and/or stability in the reinforcing properties of functions over time, and/or in relation to onset vs. maintenance. The findings highlight the utility of positioning self-harm maintenance within an affect regulatory framework of NSSH (Chapman et al., 2006; Andover and Morris, 2014; Wolff et al., 2019), that is, underlying affective difficulties and affect-laden reasons keep the self-harm going from adolescence to adulthood. If intrapersonal functions represent greater risk over time then improvements in affect regulation skills and strategies could lead to the cessation of NSSH. This was evidenced by Whitlock et al. (2015), though cessation has also been attributed also to improvements in interpersonal relationships (Tatnell et al., 2014; Whitlock et al., 2015). It is important to further understand however, whether in the context of NSSH, interpersonal relationships matter *via* their impact on emotion (e.g., Snir et al., 2018).

In contrast, the notion that *continued* engagement in self-harm occurs because normal interpersonal functions/communication methods continue to fail (the “cry of pain” model; Nock, 2008) is not supported by our data, yet it is clear that interpersonal functions are crucial to understand. During adolescence they may play a more prominent role in self-harm initiation, whereas in adulthood they are less common and do not occur without the presence of intrapersonal functions. Even though intrapersonal functions maintain the self-harm, for some people functions may change and evolve (e.g., from intrapersonal to both intrapersonal and interpersonal). We recommend therefore that clinician assessment of self-harm should repeatedly enquire about all functions, and this may give some indication of the likelihood of future repetition and suicide risk. Moreover, therapeutic interventions such as Dialectical behavior Therapy (DBT; Linehan, 2015) that develop emotion regulation skills along with interpersonal communication skills may be most effective. DBT has already shown to produce simultaneous reductions in self-harm and suicidal behavior (Linehan et al., 2006; Stanley et al., 2007).

## Data Availability Statement

The datasets presented in this article are not readily available because, there are charges for accessing ALSPAC data. Requests to access the datasets should be directed to http://www.bristol.ac.uk/alspac/researchers/access/.

## Ethics Statement

The studies involving human participants were reviewed and approved by Ethical approval for the study was obtained from the ALSPAC Ethics and Law Committee and the Local Research Ethics Committees. Written informed consent to participate in this study was provided by the participants' legal guardian/next of kin.

## Author Contributions

KG led the conceptualization of the paper and study and drafted the original manuscript. BM and EP contributed to conceptualization, design, led on the methods, approach to analysis, with KG, BM, and EP agreeing the final analytic strategy, and conducted the statistical analyses. All authors contributed to drafts of the manuscript, including approving the final version of the manuscript.

## Conflict of Interest

The authors declare that the research was conducted in the absence of any commercial or financial relationships that could be construed as a potential conflict of interest.
