# Supplementary material for: Intrapersonal and Interpersonal Functions as Pathways to Future Self-Harm Repetition and Suicide Attempts
Source: Front Psychol. 2021 Jul 19;12:688472. doi: 10.3389/fpsyg.2021.688472 (PMC8326376; doi:10.3389/fpsyg.2021.688472)
Supplement: Supplementary file 1 [file Data_Sheet_1.PDF]

Supplementary Table 1:

Self-harm functions at age 16 and 21 years (reported for the last self-harm episode)

	Functions at 16<br>N=528<br>N (%)	Functions at 21<br>N=59<br>N (%)
**Intrapersonal functions**		
[1]To punish self	178 (69.0)	35 (59.3)
[1] Relief from terrible state of mind	324 (61.4)	53 (89.8)
Anger/frustration	45 (8.5)	<5 (<8.5%)
Release feeling/coping mechanism	10 (1.9)	<5 (<8.5%)
Curiosity/friend did it	15 (2.8)	<5 (<8.5%)
Stress	<5 (<1%)	<5 (<8.5%)
Boredom	5 (1.0)	N/A
Life problems	9 (1.7)	<5 (<8.5%)
To feel in control	<5 (<1%)	<5 (<8.5%)
To take mind off something	8 (1.5)	<5 (<8.5%)
To feel pain	<5(<1%)	<5 (<8.5%)
Negative feelings towards self	17 (3.2)	<5 (<8.5%)
Felt down/depressed	25 (4.7)	<5 (<8.5%)
Wanted to get away	<5 (<1%)	N/A
Wanted to feel alive	6 (1.1)	<5 (<8.5%)
It felt good	11 (2.1)	<5 (<8.5%)
Felt like it	8 (1.5)	N/A
[1]To die (not at 16)	N/A	14 (23.7)
**Interpersonal functions**		
[1]Show how desperate	123 (23.3)	19 (32.2)
[1]To frighten someone	32 (6.1)	6 (10.2)
For attention/to show how I am feeling	<5 (<1%)	<5 (<8.5%)
So as not to hurt others	N/A	<5 (<8.5%)

Notes:

Participants could endorse multiple functions.

Cell counts less than 5 cannot be shown.

Participants who reported ‘I wanted to die’ as a reason for self-harm at age 16 years were excluded

N/A: not reported as a reason at this time point.

[1] refers to response options that were provided to participants. All other responses were generated from free text to an ‘other’ response option

Supplementary Table 2:

Imputed vs complete case data

	Complete case (adjusted)[1]		Imputed data (adjusted)	
	**OR (95%CI)**	**P value**	**OR (95%CI)**	**P value**
**Repeat self-harm**				
Total functions	1.31 (0.91, 1.89)	0.143	1.40 (1.07, 1.84)	0.015
Intrapersonal functions	1.41 (0.90, 2.21)	0.130	1.46 (1.06, 2.01)	0.021
Interpersonal functions	1.14 (0.62, 2.10)	0.665	1.30 (0.85, 2.01)	0.230
**New onset suicidal self-harm**				
Total functions	1.25 (0.84, 1.87)	0.277	1.28 (0.95, 1.74)	0.108
Intrapersonal functions	1.34 (0.82, 2.19)	0.244	1.36 (0.94, 1.97)	0.101
Interpersonal functions	1.10 (0.56, 2.14)	0.783	1.15 (0.73, 1.82)	0.548

[1] *Complete case N = 198 for repeat self-harm and 192 for suicidal self-harm*
